# The intestinal mucosa-associated microbiota in IBD-associated arthritis displays lower relative abundance of *Roseburia intestinalis*

**DOI:** 10.1080/19490976.2025.2505114

**Published:** 2025-05-18

**Authors:** Madeline Alizadeh, Uni Wong, Bernadette C. Siaton, Michael T. France, Seema A. Patil, Lauren George, Dania Hudhud, Kiran Motwani, William H. Scott, Jean-Pierre Raufman, Erik C. von Rosenvinge, Raymond K. Cross, Jacques Ravel

**Affiliations:** a Institute for Genome Sciences, University of Maryland School of Medicine, Baltimore, MD, USA; b Division of Gastroenterology and Hepatology, Department of Medicine, University of Maryland School of Medicine, Baltimore, MD, USA; c Department of Veterans Affairs, Veterans Affairs Maryland Health Care System, Baltimore, MD, USA; d Division of Rheumatology and Clinical Immunology, Department of Medicine, University of Maryland School of Medicine, Baltimore, MD, USA; e Department of Microbiology & Immunology, University of Maryland School of Medicine, Baltimore, MD, USA

**Keywords:** Spondyloarthritis, mucosal gut microbiome, enteropathic arthritis, Crohn’s disease, ulcerative colitis, IBD-associated arthritis

## Abstract

The most common extra-intestinal manifestation (EIM) of inflammatory bowel disease (IBD), IBD-associated arthritis (IAA), occurs in 25–40% of patients and can be debilitating. In IBD, mucosal and stool microbiota richness is decreased, and compositional changes can precede or accompany disease onset. Likewise, spondyloarthritides are associated with altered gut microbiota, with overlapping bacterial signatures observed in IBD, suggesting key shared microbial factors are involved in both conditions. Much has been learned about the role of the intestinal microbiome in IBD, but less is known regarding its role in IAA. To address this knowledge gap, we analyzed the mucosa-associated intestinal microbiota of participants enrolled in the LOCATION-IBD cohort. Microbiota composition was established using 16S rRNA gene amplicon sequencing of intestinal biopsy samples taken from participants with IBD, with or without arthropathy. Microbiota samples clustered predominantly by participant, and similar taxa were present across the colon. The mucosal intestinal microbiota of females with IAA displayed a lower relative abundance of *R. intestinalis*, while males with IAA had a higher relative abundance of *Corynebacterium*, even when controlling for IBD-type, whether samples were taken from a site of inflammation and intestinal location. These findings indicate the mucosa-associated intestinal microbiota is associated with IAA in a sex-specific manner.

## Introduction

Inflammatory bowel disease (IBD), which includes Crohn’s disease (CD) and ulcerative colitis (UC), is increasingly common in the United States and worldwide.^1,2^ Extra-intestinal manifestations (EIMs) of IBD occur frequently in IBD patients, negatively impact quality of life, and are often challenging to treat.^3^ The musculoskeletal, dermatologic, ocular, and hepatobiliary systems are most frequently affected;^4^ by far, the most common EIM is IBD-associated arthritis (IAA), a form of spondyloarthritis (SpA).^5^ Known risk factors for both peripheral and axial IAA include female sex, CD, right-sided colon involvement, and other SpA-related risk factors such as human leukocyte antigen (HLA)-B27 positivity, psoriasis, and presence of other EIMs (*e.g.*, uveitis).^4,6–8^ Although anywhere from 25% to 40% of individuals with IBD are affected by IAA,^9^ much remains to be learned regarding its etiopathogenesis. Part of the challenge lies in the fact that joint-related disease activity does not always correlate with intestinal disease activity,^10,11^ suggesting that while these conditions are highly related, IBD-associated SpA is a distinct disease entity.

The intestinal microbiota has long been speculated to play a key role in the development and progression of both IBD and SpA.^3,12–16^ Multiple bacteria are differentially abundant in both conditions compared to healthy controls, including the genera *Dialister, Ruminococcus, Faecalibacterium, Roseburia, Akkermansia*, *Fusicatenibacter*, and *Enterococcus*, among others.^12,17–19^ However, not all associations overlap, and some bacteria display discordant biosignatures.^12^ Studies looking at IAA in stool have found a decreased abundance of *Bifidobacterium* and lactobacilli^20^ and an increased abundance of *Enterococcus*,^21^ *Staphylococcus*, and *Proteus*.^20^ However, most studies were performed using stool samples that approximate the bacterial composition and abundance of the entire intestinal lining, failing to accurately estimate the bacteria adherent to the gut mucosal tissue at specific intestinal locations. Mucosa-based studies may identify localized compositional changes associated with location-specific inflammation and the resultant impact on barrier integrity^22^ and can do so earlier in the disease than stool-based studies, thus making them more diagnostically valuable.^23^ Barrier disruption allows metabolites to circulate in the bloodstream, leading to systemic involvement observed in spondyloarthropathy. However, despite increasing interest in studying the mucosa-associated intestinal microbiota,^24,25^ few studies have characterized the mucosal intestinal microbiota in IBD,^25–27^ and even fewer in IAA, where the largest number of individuals sampled in a study has been five with axial IAA.^28^

To address these gaps in knowledge and better understand the relationship between the intestinal mucosal microbiota and IAA, we performed an analysis of the microbiota of intestinal biopsy samples collected from multiple locations in the lower GI tract, including healthy tissue, sites of active inflammation, and healthy-appearing tissue adjacent to areas of inflammation, from participants in the LOCATION-IBD cohort.^29^ Incorporating clinical data and phenotyping, we assessed the composition and structure of the mucosa-associated intestinal microbiota in a location- and disease-specific manner in participants with and without IAA. Our findings lay the groundwork for novel hypotheses regarding the contribution of intestinal microbiota to disease, including that the presence of *Roseburia intestinalis* protects against IAA in females with IBD. In contrast, *Corynebacterium* presence contributes to the development of IAA in males.

## Materials and methods

### Participant, sample, and metadata collection

One hundred eighty-two adults with IBD, with or without IAA, were enrolled in the LOCATION-IBD cohort.^29^ The study protocol and the cohort were previously described,^29^ including enrollment protocols, inclusion and exclusion criteria, and patient stratification; notably, patients with antibiotic exposure in the prior 4 weeks were excluded. Briefly, participants were enrolled from the University of Maryland IBD clinic and our associated Baltimore Veterans Affairs Medical Center IBD clinic. Intestinal mucosal biopsies (approximately 8–10 mg each) were collected from the terminal ileum (TI), hepatic flexure (HF, *i.e*., proximal colon, PC), and distal descending colon (DDC) during clinically indicated colonoscopy.^29^ Sampling locations were chosen as follows. TI and DDC are most frequently involved in CD and UC, respectively. Ileal involvement is observed in up to 70–80% of individuals with CD.^30^ Distal colon involvement just beyond the rectum is involved for 75–80+% of individuals with UC seen at our institution. HF was additionally sampled based on previous work from our lab^7^ which has shown right-sided colon involvement is more frequently associated with many EIMs, including IAA. Areas of inflammation as determined by the endoscopists included tissue with features of erythema, erosion, and/or ulceration; when present, these areas and adjacent healthy tissue were both biopsied. Biopsy samples were stored in 1 ml of DNA/RNA Shield (ZYMO Research) for microbiota analysis and kept at −80°C until DNA extraction. Clinical, demographic, and patient-reported outcome (PRO) data were collected using a combination of chart review, participant interview, and REDCap survey.

### DNA extraction and library preparation

Samples (1 ml) were homogenized using bead-beating (4.0 m/s, 20s, mpbio FastPrep-24), performed with 0.1 mm silicon beads (Lysing Matrix B 2 mL RNAse/DNAse free tubes, mpbio, San Diego, CA, USA); DNA was extracted from 350 µL of homogenized tissue using the NEB Monarch Genomic DNA Purification Kit (NEB, Ipswich, MA, USA), following the animal tissue protocol, per manufacturer’s instructions. Bacterial DNA was enriched using the NEBNext Microbiome DNA Enrichment Kit (NEB, Ipswich, MA, USA), without modification of the manufacturer’s protocol. DNA was eluted into 50 µl of TE buffer and stored frozen at −80°C until use.

### 16S rRNA gene V3V4 amplicon sequencing

The composition of the mucosal intestinal microbiota was determined using amplicon sequencing of the V3V4 region of the 16S rRNA gene, as described by Holm et al.,^31^ except that unique dual indices were used. Amplicon libraries were sequenced on an Illumina NextSeq 1000 instrument at a targeted depth of 50,000 paired-end reads per sample, using the PE300 protocol (600 cycle), at Maryland Genomics, Institute for Genome Sciences, University of Maryland School of Medicine. Negative (extraction and PCR, using the same protocols described above) and positive control samples (ZymoBIOMICS Microbial Community DNA Standard, ZYMO Research) were included and sequenced using the same procedures.

### Data processing, filtering, and normalization

Paired-end reads were processed using DADA2^32^ to assemble amplicon sequences, remove chimeric sequences and sequencing errors, and identify amplicon sequence variants (ASVs). ASV taxonomic assignments were performed using the RDP classifier^33^ trained on the SILVA database^34^ (release 138.1), and ASVs with similar taxonomic assignments were merged. Samples with fewer than 5,000 sequences were removed from the dataset (*n* = 71 removed, 490 remaining). Further processing involved removing all taxa and sequences assigned as “unknown”, “eukaryote”, and “chloroplast”, leaving a total of 2,238 bacterial taxa and a mean of 78,405 sequences per sample (median 68,219, IQR = [31,320, 112, 770], range = [5,224, 264,473]). Bacterial taxa were filtered such that those with a study-wide mean relative abundance of less than 5 × 10^−5^ were removed, leaving 384 taxa. The minimum count for any remaining sample following these preprocessing steps was 4,999 sequences per sample, with a mean of 78,828 sequences (median 67,772, IQR = [31,021, 111,817], range = [4,999, 260,520], *n* = 490, Supplemental Figure S1; representing a mean of 2.85 biopsies, a median of 3 biopsies, IQR [2,3], range [1,6], per participant). Preprocessing resulted in the generation of a final table containing sequence counts for each bacterial taxon for each sample (Supplemental Table S1), used for all statistical analyses described below.

### Demographic analysis of the cohort with samples used in the analyses

Differences in demographic variables between individuals with and without IAA and those with joint pain of unclear etiology were assessed using Fisher’s Exact test for categorical variables and ANOVA for quantitative variables.

### Comparative analysis of the mucosal intestinal microbiota composition

Principal component analysis (PCA) of the taxa relative abundance data was performed, and the 1st and 2nd PCs were plotted and color-coded based on multiple clinical factors, including IBD type, sex, whether samples were taken from inflamed tissue, sample location origin, and joint EIMs. PCA plots were visualized using the “stats” and “ggbiplot” packages in R.^35^ Stacked bar plots displaying taxa relative abundance were generated for each intestinal biopsy location (TI, HF, DDC), as well as for all 490 samples in the cohort. Relative abundance of the 20 most highly abundant taxa in each category and an “other” category comprised of the sum of relative abundances of all other taxa were plotted. Spearman test was used to assess differences in the relative abundance of all 384 taxa between each site due to the results of the Shapiro–Wilk test on relative abundance data, which provided a p-value of <2.2 × 10^−16^. Dendrograms displaying hierarchical clustering of samples were produced based on Euclidean distances and Ward linkage. Zicoseq was used to assess differences in composition between intestinal locations (TI, HF, DDC) while controlling for participants.

### Analysis of alpha and beta diversity

Alpha diversity was calculated using Shannon indices using the *phyloseq* package in R for each sample, and values were compared based on IBD type, sex, whether samples were taken from inflamed tissue, whether inflammation was present on endoscopic exam, sample location, and joint EIMs. Beta diversity was calculated using Jensen Shannon Divergences (JSDs) via the *phyloseq* package in R comparing locations, specifically between locations within participants and across locations in the cohort. Individual location–location comparisons (*n* = 524) were averaged to generate a single composite mean value for JSD for each participant. Mean JSDs were compared based on whether inflammation was present on endoscopic exam, disease localization, IBD type (including CD, UC, and IBD-type undetermined, *i.e.*, IC), joint EIMs, and biologic medication use. Statistical significances between univariate factors between groups were evaluated using Welch’s t-test for two-group comparisons, or one-way ANOVAs for multi-group comparisons with post-hoc Tukey analysis, using the *stats* package in R.^35^

### Comparison of taxa in those with and without joint EIMs and across IBD types

ALDEx2 t-test function was employed to identify differences between those with and without joint EIMs, excluding those with joint pain of unclear etiology (*n* = 438 total remaining), and Benjamini–Hochberg (BH) corrected q-values were calculated. A cutoff significance level of 0.100 for calculated q-values of either Welch’s t-test or Wilcoxon Rank test was used to identify taxa with significant associations. A series of multiple logistic multivariate models using generalized linear models (GLMs) with a binomial distribution using the *stats* package^35^ in R were generated to describe the relationships between central log ratio (CLR) transformed individual bacteria relative abundance and joint EIMs while controlling for IBD type, whether a sample was obtained from a site of inflammation, and sampling location, with a significance level of 0.100. These models were validated using mixed GLMs with participant ID as a random effect to account for participant representation in multiple biopsies, using the *lme4* package in R. The same modeling approach was employed to describe the relationship between joint EIMs, CLR-transformed bacterial taxa relative abundance, sex, IBD type, and the relationship between CLR-transformed bacterial taxa relative abundance and sex, and CLR-transformed bacterial taxa relative abundance and IBD type. A mixed GLM (with participant ID as a random effect) was used to model the relationship between joint EIMs and *R. intestinalis* presence/absence, IBD-type, sex, and the interaction between bacterial presence/absence and sex. For those with joint EIMs, the relationship between active joint disease, sex, and CLR-transformed bacterial taxa relative abundance was assessed using one-way ANOVA tests for multi-group comparisons (males and females with and without IAA), with post-hoc Tukey analysis, due to sample size preventing more complex models. A similar mixed GLM (with participant ID as a random effect) was used to assess, as a categorical variable, *R. intestinalis* relative abundance being in the 4^th^ quartile across all samples in place of *R. intestinalis* presence/absence. Finally, a mixed GLM (with participant ID as a random effect) was employed to describe the relationship between IBD type and bacterial taxa relative abundance, sex, age, presence or absence of joint EIMs, and the relationship between bacterial taxa relative abundance and sex. A Q-value correction was performed on all p-values generated via the GLMs developed using similar base equations via False Discovery Rates to account for multiple comparisons.

### Software and figure generation

All analyses were performed with R version 4.3.1.^35^ All figures were edited in Inkscape to adjust figure titles, text, fonts, and text size and add sub-labels where appropriate. The content of the figures was unaltered and can be recreated using the code provided in the data availability statement.

## Results

### Study subject demographics

Demographics of the sub-cohort with samples remaining in the dataset used for analyses (*N* = 172) were assessed in a sensitivity analysis and are described in Table 1. Similar to the main cohort,^29^ those with joint EIMs were still more likely to be female (p-value = 0.015) and have CD (p-value = 0.051), but were not different with regard to age, race, IBD type/sex sub-stratification, CD location or behavior, or extent of disease of UC. Due to the low number of participants with imaging-confirmed axial IAA (*n* = 2 with isolated axial, *n* = 1 with axial and peripheral disease), participants with axial and peripheral IAA were condensed into one group (those with IAA). Because IBD type and sex are previously described to be associated with gut microbiota composition, differences in both were assessed and, when applicable, controlled for in analyses.

**Table 1.** Demographics of the LOCATION-IBD cohort participants included in the microbiome analysis and sub-groups of participants with joint EIMs (JEIMs), no JEIMs, and joint pain of unclear etiology.

Factor	Sub-Cohort* (n = 172)	JEIMs (n = 51)	No JEIMs (n = 103)	Unclear etiology (n = 18)	p-value^#^
Sex (female)	84 (48.8%)	32 (59.3%)	41 (39.8%)	11 (61.1%)	0.015
Age (mean, median, IQR, range)	41.6 39.5 [31,51] [19,77]	42.5 42 [42.5,50] [20,73]	40.6 38 [30,51.3] [19,71]	44.3 46 [30.5,52.8] [21,77]	0.475
Self-described race −White −Black −Asian −Mixed/Other	129 (75.0%) 31 (18.0%) 8 (4.7%) 4 (2.3%)	36 (70.6%) 11 (21.6%) 1 (2.0%) 3 (5.9%)	78 (75.7%) 17 (16.5%) 7 (6.8%) 1 (1.0%)	15 (83.3%) 3 (16.7%) 0 (0%) 0 (0%)	0.406
IBD type −CD −UC −IC	116 (67.4%) 46 (26.7%) 10 (5.8%)	39 (76.5%) 7 (13.7%) 5 (9.8%)	64 (62.1%) 35 (34.0%) 4 (3.9%)	13 (72.2%) 4 (22.2%) 1 (5.6%)	0.051
IBD type stratified by sex (female) −CD −UC −IC	60 (71.4%) 19 (22.6%) 5 (5.9%)	25 (78.1%) 4 (12.5%) 3 (9.4%)	27 (65.9%) 13 (31.7%) 1 (2.4%)	8 (72.7%) 2 (18.2%) 1 (9.1%)	0.219
Disease extent (UC) −Proctitis −Proctosigmoiditis/Left-sided −Extensive/Pancolitis −Unknown	7 (15.2%) 11 (23.9%) 27 (58.7%) 1 (2.2%)	0 (0%) 3 (42.9%) 3 (42.9%) 1 (14.3%)	7 (20.0%) 6 (17.1%) 22 (62.9%) 0 (0%)	0 (0%) 2 (50.0%) 2 (50.0%) 0 (0%)	0.1105
Disease location (CD) −Ileal −Colonic −Ileocolonic	45 (38.8%) 16 (13.8%) 55 (47.4%)	13 (33.3%) 8 (20.5%) 18 (46.2%)	29 (45.3%) 6 (9.4%) 29 (45.3%)	3 (23.1%) 2 (15.4%) 8 (61.5%)	0.311
Disease phenotype (CD) −Inflammatory −Stricturing −Penetrating −Stricturing & Penetrating	43 (37.1%) 38 (32.8%) 28 (24.1%) 7 (6.0%)	15 (38.5%) 13 (33.3%) 9 (23.1%) 2 (5.1%)	24 (37.5%) 21 (32.8%) 15 (23.4%) 4 (6.3%)	4 (30.8%) 4 (30.8%) 4 (30.8%) 1 (7.7%)	0.996
Inflammation present on endoscopic exam	78 (45.1%)	23 (45.1%)	47 (45.2%)	8 (44.4%)	≥0.999

*Subset of participants from the original cohort with biopsy samples (n = 490 samples) included in the final analysis.

^#^p-value represents results of ANOVA for quantitative variables, Fisher’s exact test for qualitative variables, comparing the three subgroups.

### The intestinal mucosal microbiota clusters predominantly by participant

**Figure 1.**
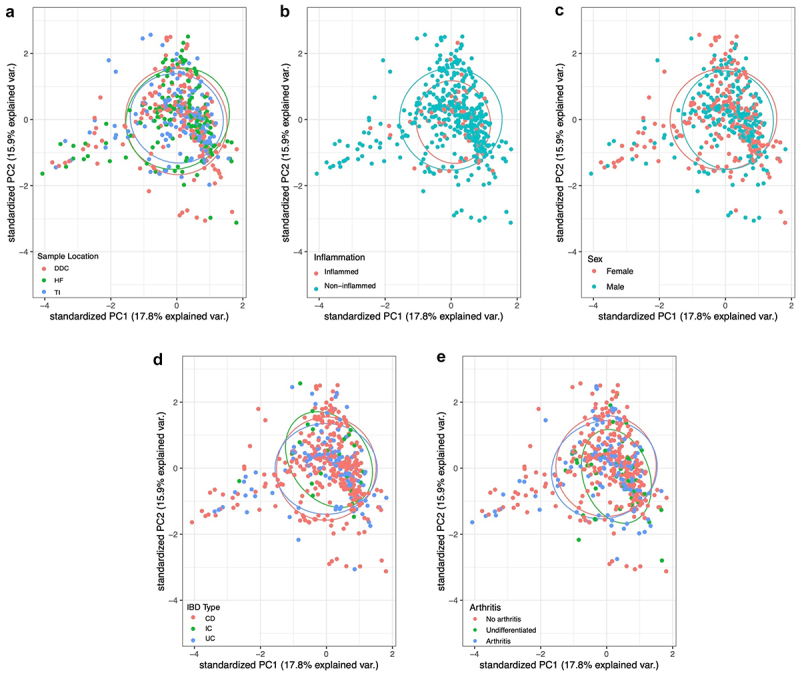
PCA based clustering of relative abundance normalized intestinal mucosal microbiota compositional data. (a) sampling locations, (b) presence and absence of inflammation at sampling site, (c) sex, (d) IBD type, and (e) joint EIM status. DDC = distal descending colon, HF = hepatic flexure, TI = terminal ileum, CD = Crohn’s disease, IC = IBD-type undetermined, UC = ulcerative colitis.

In light of the known differences in the fecal microbiota between CD vs UC, and the physiological differences in the mucosa of proximal and distal portions of the colon,^36,37^ we compared the relative abundance of the intestinal mucosa-associated microbiota using principal component analysis. We explored multiple potentially explanatory factors, including IBD type, history of joint EIMs, participant sex, sampling location (TI, HF, DDC), and whether they were obtained from inflamed tissue (Figure 1). Sample bacterial compositional profiles did not cluster based on any of the evaluated factors, with a nearly complete overlap of the 95% confidence ellipsoid for each factor. Instead, samples clustered most closely based on the participant from whom they were obtained, with less variation observed within rather than across participants (Supplemental Figures S2 and S3).

**Figure 2.**
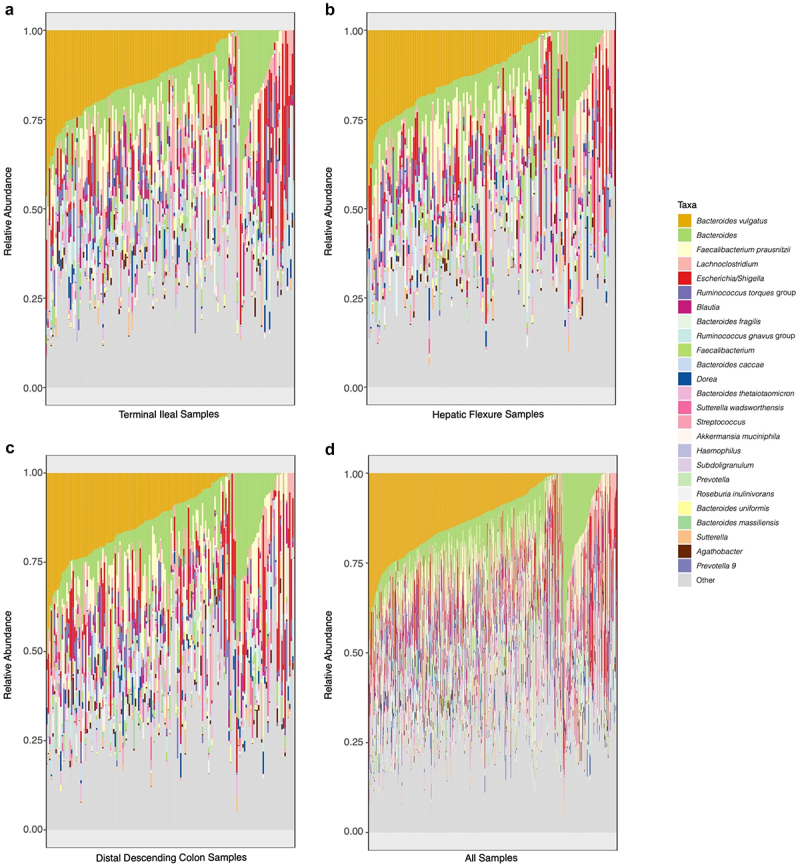
Stacked bar plots of bacterial taxa relative abundance in the intestinal mucosal microbiota of the TI (a), HF (b), and DDC (c), as well in all samples collected (d).

Beta diversity was used to assess the similarity of the composition of the intestinal mucosal microbiota. Mean Jenson-Shannon divergence (JSD) across all samples was 0.4002 (median = 0.3938, IQR = [0.3145, 0.4824], range = [0.0028, 0.6888]; Supplemental Figure S4). We then compared the relative abundance of the 20 most abundant bacterial taxa in each location. These bacterial taxa accounted for 63.7% of the total abundance in TI samples, 61.9% of HF samples, 63.0% of DDC samples, and 62.6% of the total abundance across all samples. *Bacteroides vulgatus* and *Bacteroides* were the first and second most prevalent bacteria, with *Faecalibacterium prausnitzii* and *Lachnoclostridium* third and fourth in TI and DDC samples, and third and fifth, respectively, in the HF samples (Figure 2). Nineteen of the 20 most abundant bacterial taxa overlapped between TI, HF, and DDC samples, and the Spearman correlation between bacterial taxa relative abundance in TI and HF samples, TI and DDC samples, and in HF and DDC samples was ≥0.9999 (p-values = <2.2 × 10^−16^ for all), indicating a high degree of congruence in both prevalence and relative abundance of bacterial taxa present in TI, HF and DDC locations. Interestingly, even when comparing all the taxa in the dataset, no bacterial taxa were more highly associated with any one location (all FDR adjusted p-values ≥0.100).

### Species richness of intestinal mucosal microbiota is higher in UC than CD or IC

**Figure 3.**
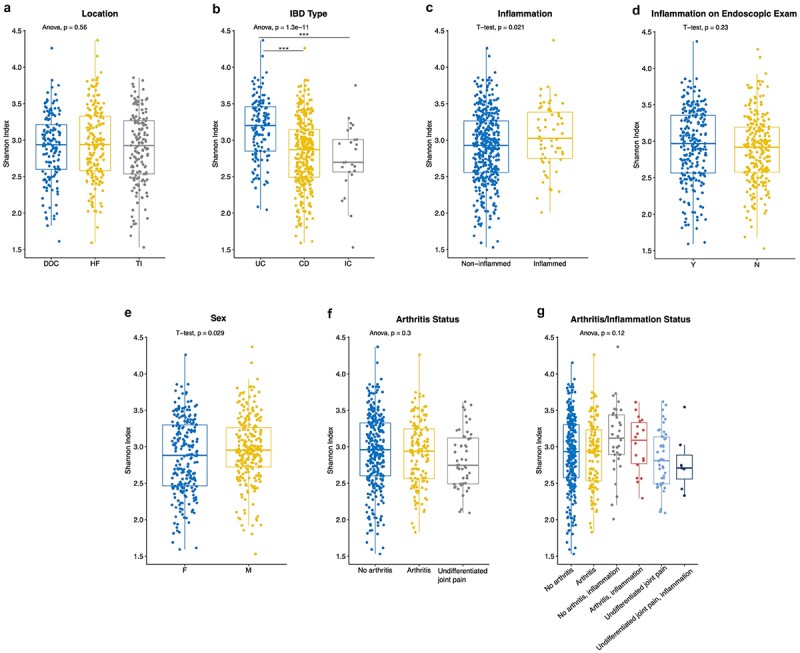
Intestinal mucosal microbiome alpha diversity comparison by sampling location (a), IBD type (b), whether or not a sample was taken from a site of inflammation (c), whether or not erythema/inflammation was present on endoscopic exam (d), sex (e), joint EIM status (f), and joint EIM status sub-stratified based on whether or not a sample was taken from a site of inflammation (g). DDC = distal descending colon, HF = Hepatic flexure, TI = Terminal ileum, CD = Crohn’s disease, IC = IBD-type undetermined, UC = Ulcerative colitis, F = Female, *M* = Male. Boxes represent the IQR. ***p ≤ 0.001.

Alpha diversity of the intestinal mucosal microbiota was compared between sampling location, IBD type, whether the sample was obtained from inflamed tissue, whether inflammation was present on an endoscopic exam, sex, history of joint EIMs, and whether the sample was obtained from a site of inflammation stratified by arthritis status (Figure 3). The mean Shannon index across all the samples was 2.92 (median = 2.94, IQR = [2.57,3.28], range = [1.53, 4.37]; Supplemental Figure S5). Alpha diversity was significantly different when IBD types were compared, with a higher mean Shannon index in UC compared to CD and IC (3.16 vs 2.83 and 2.75, respectively; ANOVA p-value = 1.33 × 10^−11^, Tukey post-hoc p-value ≤ 10^−8^ and 0.0002, respectively; Figure 3b). Additionally, differences in alpha diversity were observed based on whether a sample was obtained from a site of inflammation (3.04 in inflamed vs 2.90 in non-inflamed samples, p-value = 0.021; Figure 3c), and sex (2.87 in females vs 2.96 in males, p-value = 0.029; Figure 3e), but not based on sampling location (Figure 3a), presence of inflammation on endoscopic examination (Figure 3d), arthritis status (Figure 3f), or arthritis/inflammation sub-stratification (Figure 3g).

### Intestinal mucosa-associated microbiota composition does not vary more across the colon in those with IBD-associated arthritis

**Figure 4.**
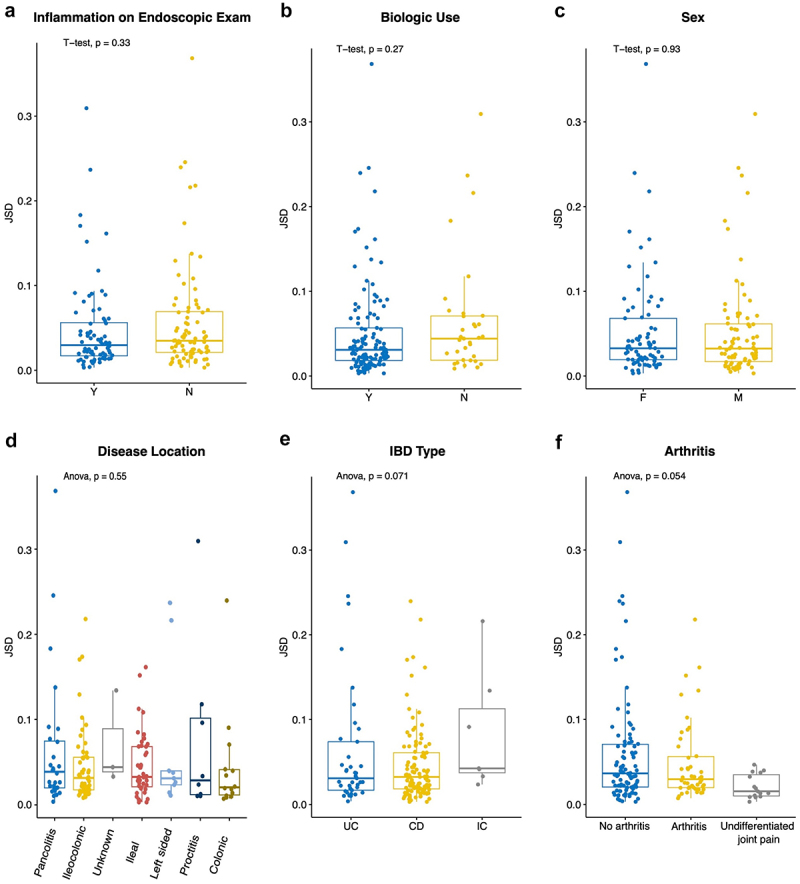
Intestinal mucosal microbiota mean beta diversity (JSD) across samples for each individual, stratified by multiple clinical factors. JSDs were calculated between each site and averaged for a given individual to assess beta diversity across samples for each participant, and values were compared based on whether or not inflammation was present on endoscopic exam (a), biological drug use (b), sex (c), extent of disease (UC, IC) or disease location (CD) (d), IBD type (e), and joint EIM status (f). CD = Crohn’s disease, IC = IBD-type undetermined, UC = Ulcerative colitis, F = Female, *M* = Male. Boxes represent the IQR.

The high degree of similarity in the composition of the intestinal mucosal microbiota between various locations in the colon led us to explore the level of inter-participant differences and whether clinical characteristics influenced this variation. We calculated the mean JSDs between each site for participants with more than one sample (*n* = 157 participants, 46 with joint EIMs, 96 without joint EIMs, and 15 with joint pain of unclear etiology; Supplemental Figure S6). The mean JSD for each participant across multiple sampling locations was low, at 0.0526 (median = 0.0328, IQR = [0.0184, 0.0620], range = [0.0031, 0.3864]). There was no difference in mean JSD for participants based on the presence or absence of inflammation on an endoscopic exam (p-value = 0.3276, Figure 4a), biologic drugs use (p-value = 0.2682, Figure 4b), sex (p-value = 0.9094, Figure 4c), or disease location (p-value = 0.3580; Figure 4d). There was a trend toward increased variability across sites in those with IC compared to UC and CD (*p*-value = 0.0710, Figure 4e), and in those with joint pain of unclear etiology compared to those with or without arthritis (*p*-value = 0.0538, Figure 4f), however, these did not reach statistical significance. Altogether, these results suggest that large compositional differences in the mucosal-associated microbiota are not contributing to disease in those with or without joint EIMs of IBD, but rather, variations in the relative abundance of a small number of bacterial taxa may be involved.

### *Several bacterial taxa relative abundances are higher in those with IBD-associated arthritis, while that of* R. intestinalis *is lower*

**Figure 5.**
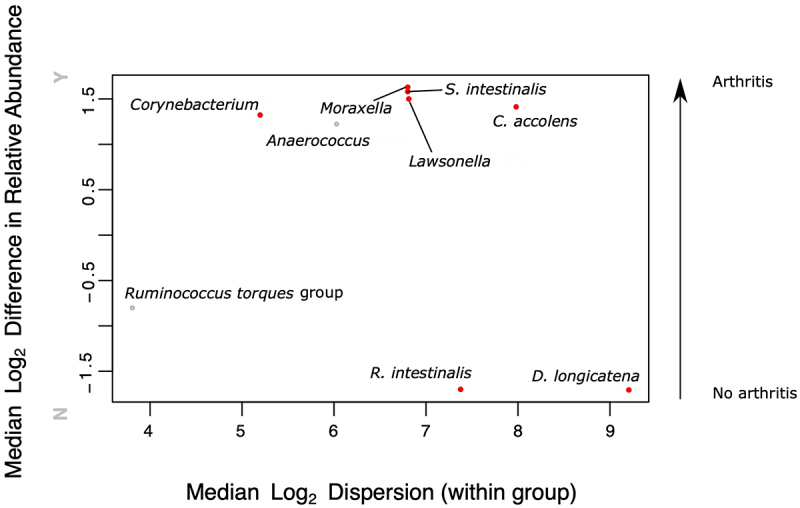
Differences in bacterial taxa relative abundance in the intestinal mucosal microbiota in those with or without joint EIMs. Univariate assessment of differences in relative abundance between bacterial taxa in those with or without joint EIMs were performed using ALDEx2. Y = IAA present, *N* = IAA absent. Red points = Welch’s t-test q-value ≤0.10, gray points = Wilcoxon rank test q-value ≤0.10 with Welch’s t-test q-value >0.10.

To identify which specific bacteria may be associated with IAA, we compared bacterial taxa relative abundance in samples from those with or without IAA (Supplemental Table S2, Figure 5). Those with IAA were more likely to display higher relative abundance of *Anaerococcus, Moraxella, Corynebacterium, Lawsonella* spp., *Sellimonas intestinalis* and *Corynebacterium accolens* (Welch’s q-values = 0.1748, 0.0637, 0.0308, 0.0906, 0.0547 and 0.0885 respectively). In contrast, those without IAA were more likely to have higher relative abundances of *R. intestinalis*, *Dorea longicatena*, and *Ruminococcus torques* group spp. (Welch’s q-values = 0.0499, 0.0321, and 0.1729, respectively; Figure 5).

Given that multiple potential confounders could significantly alter bacterial relative abundances and thus skew the comparative analyses between groups, we generated a series of GLMs assessing joint EIM status as a function of central log ratio (CLR) transformed bacterial relative abundance while controlling for IBD type, sampling location, and whether a sample was from inflamed tissue (Supplemental Table S3). In agreement with previous results, the higher *R. intestinalis* abundance corresponded to a decrease in the likelihood of joint EIMs (q-value = 0.0058). In contrast, higher *Butyricicoccus, Frisinginococcus*, and *Roseburia hominis* increased abundance corresponded to higher odds of having joint EIMs (q-values = 0.0148, 0.0652, and 0.0472, respectively). Additionally, *Laedolimicola ammoniilytica (*identified as GCA.900066575) relative abundance was found to be lower in those with IAA (q-value = 0.0148), with each one-point increase in relative abundance corresponding to a 0.534 decreased likelihood of joint EIMs. A second set of mixed GLMs accounting for participant ID as random error produced almost identical values but were not used initially due to an incalculable confidence interval.

**Figure 6.**
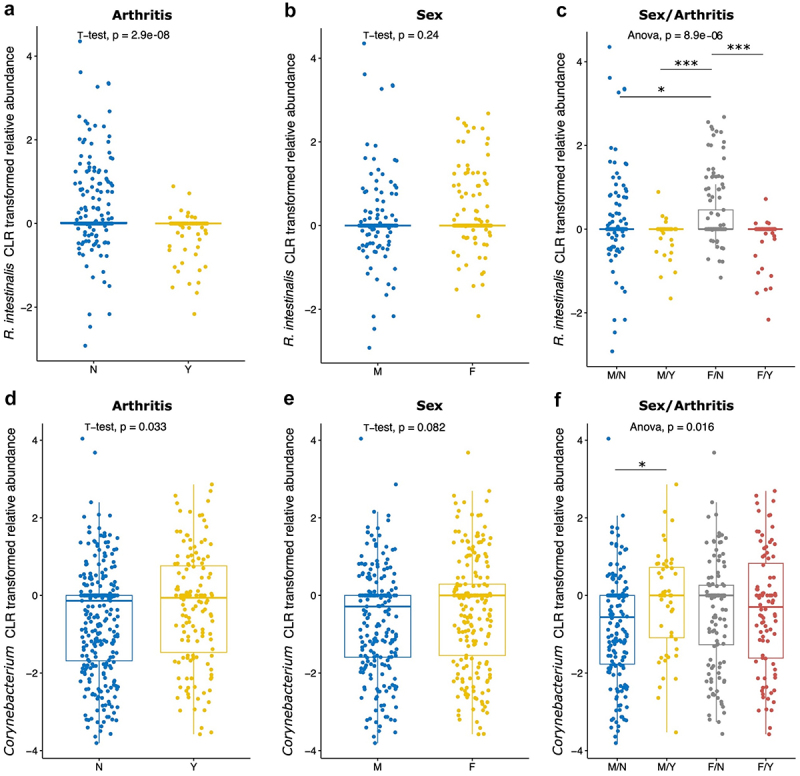
Relative abundance of *R. intestinalis* and *Corynebacterium* by sex, joint EIM status, and joint EIM status sub-stratified by sex. CLR transformed relative abundance values were compared, and t-test was used for 2-group comparisons of *R. intestinalis* and sex (a), *R. intestinalis* and joint EIM status (b), *Corynebacterium* and sex (d), and *Corynebacterium* and joint EIM status (e), while one-way ANOVA was used for multi-group comparisons with *R. intestinalis* (c) and *Corynebacterium* (f). *M* = male, F = female, Y = arthritis, *N* = no arthritis. *p ≤ 0.05, ***p ≤ 0.001. Boxes represent the IQR.

Because no association was observed between the CLR-transformed bacterial relative abundances and both sampling location and inflammation, and both those factors and joint EIMs, these were removed from the models. Models were generated to evaluate the association of joint EIMs with the bacterial taxa identified above (using both univariate and multivariate models), sex, IBD type, and the relationship between those bacterial taxa and sex, and those bacterial taxa and IBD type (Figure 6; Supplemental Table S4). Similar results between the mixed and original models were observed, indicating that differences in individual bacterial taxa in those with and without joint EIMs do not vary significantly when accounting for participant variation. In almost all models, UC and male sex were associated with a lower likelihood of joint EIMs, while the interaction between IBD type and bacterial taxa relative abundance showed no association with EIMs, with the exception of the model assessing *Corynebacterium*. The interactions between *R. intestinalis, Corynebacterium*, *Moraxella*, and *Butyricicoccus* relative abundances and sex display a significant association with joint EIMs; however, only relative abundances of *R. intestinalis* alone were also significantly associated with joint EIMs (*i.e*. associated regardless of sex).

Specifically, *R. intestinalis* relative abundance maintained a significant individual association with joint EIMs and was lower in females with joint EIMs (p-value = 8.9 × 10^−6^; Figure 6c). On the other hand, *Corynebacterium* relative abundance was higher in males with joint EIMs (p-value = 0.016; Figure 6f), while *Moraxella* relative abundance was lower in males with joint EIMs. *Butyricicoccus* relative abundance was higher in males with joint EIMs but trended higher in females without joint EIMs, though this association was not statistically significant.

**Figure 7.**
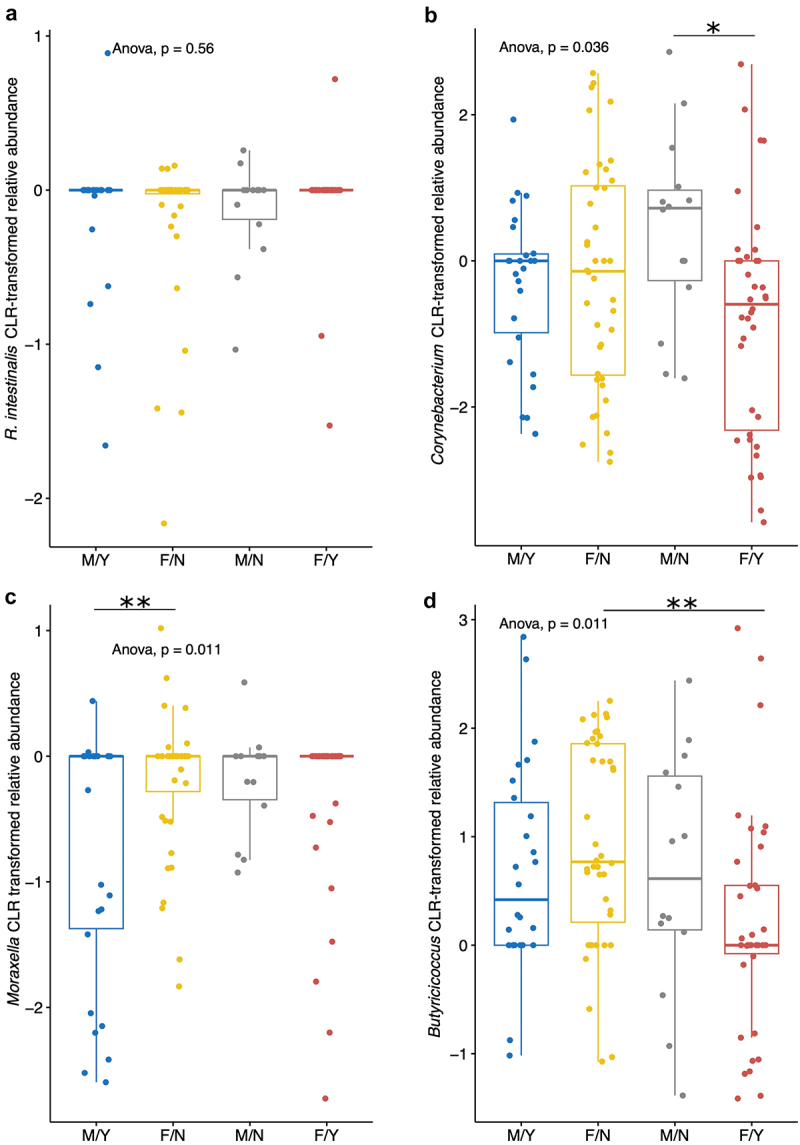
Relative abundance of *R. intestinalis* and *Corynebacterium* by sex and joint disease activity status. CLR-transformed relative abundance values were compared, and one-way ANOVA was used for comparisons of *R. intestinalis* (a), *Corynebacterium* (b), *Moraxella* (c), and *Butyricicoccus* (d), in participants sub-stratified by sex and joint disease activity (active vs inactive) for those with joint EIMs (*n* = 51 total, 6 males without (M/N), 9 males with (M/Y), 15 females without (F/Y), and 14 females with (F/Y) active joint disease). *M* = male, F = female, Y = active joint EIMs, *N* = inactive joint EIMs. *p ≤ 0.05, **p ≤ 0.01. Boxes represent the IQR.

For those with joint EIMs, we performed a sub-analysis comparing those with vs without active joint disease. Interestingly, *R. intestinalis* abundance did not significantly differ between participants based on joint disease activity (Figure 7a). *Corynebacterium* relative abundance was significantly lower in females compared to males without active joint disease (Tukey post-hoc p-value = 0.0299; Figure 7b), while *Moraxella* relative abundance was significantly lower in males with active joint disease than females without and trended toward being lower than males without and females with active joint disease (Tukey post-hoc p-values = 0.0097, 0.0750, and 0.0536, respectively; Figure 7c). *Butyricicoccus* relative abundance was lower in females with active joint disease than those without (Tukey post-hoc p-value = 0.0057; Figure 7d).

A mixed GLM evaluating the association between joint EIMs and *R. intestinalis* relative abundance in the 4^th^ quartile across all samples was generated to further characterize whether a threshold for bacterial abundance may be associated with a higher likelihood of joint EIMs. Again, UC and male sex were associated with a lower likelihood of joint EIMs (Supplemental Table S5). However, for females, being in the 4^th^ quartile for *R. intestinalis* relative abundance was found to be modestly protective and associated with a lower likelihood of joint EIMs, further validating the results described above. Similar models were generated for *Corynebacterium*, but the taxa relative abundances and sex interaction were not statistically significant.

### Relative abundance of bacterial taxa associated with IBD type are distinct from those associated with joint EIMs

Because CD is associated with joint EIMs, and IBD type is known to be associated with the differential relative abundance of various bacterial taxa, we queried which bacterial taxa relative abundance was associated with IBD types while controlling for potentially confounding factors. Multivariate GLMs were constructed to describe the relationship between IBD type (CD and UC only) and bacterial taxa relative abundance. The models controlled for joint EIMs (higher prevalence in CD), sex (female skewed in CD, though not statistically significant), the interaction between sex and bacterial taxa relative abundance, and age (lower in CD) (Supplemental Table S6). Overall, the relative abundance of 43 bacterial taxa were significantly associated (based on q-value) with IBD type, including *Subdoligranulum, Dorea longicatena*, *Dorea formicigenerans, Coprococcus catus, Alistipes finegoldii*, and *Bacteroides ovatus*, which were more likely (OR > 2) to have a higher relative abundance in those with CD, and *L. ammoniilytica, Eisenbergiella tayi, Merdibacter, Hungatella*, and *Adlercreutzia equolifaciens*, which were more likely to have a higher relative abundance (OR < 0.5, OR for CD relative to UC) in those with UC. Only *Ruminococcus torques* group and *Dorea longicatena* had relative abundances associated with both IBD types and joint EIMs, significantly more likely to be higher in those with CD. These results indicate that the relationships between specific bacterial taxa relative abundances and joint EIMs are not driven exclusively by IBD type.

## Discussion

In this study, we report on the composition of the intestinal mucosal microbiota in patients with IBD, with and without IAA. We found that the overall bacterial composition was individualized and that no specific structures of the microbiota or bacteria were associated with the mucosal locations sampled (TI, HF, and DDC), even when evaluating all taxa in the dataset. This finding agrees with previously reported findings from Torres et al., who reported that regardless of disease status, the structure of the intestinal mucosal microbiota is relatively similar between the terminal ileum, right colon, and left colon when comparing IBD, IBD with concomitant primary sclerosing cholangitis (PSC), and healthy controls.^27^ This finding was corroborated by Vaga et al., who only observed subtle differences in the compositional structure of the microbiota across the colon within healthy individuals.^36^

Interestingly, stratifying based on overall composition by IBD type, sites of inflammation, or IAA status did not reveal further associations. The inability to cluster samples using IBD type was intriguing given that stool microbiota composition can be used to differentiate between IBD type and disease location.^23,38^ The intestinal mucosa-associated microbiota is enriched for several taxa and depleted of others, and this difference in composition likely contributes to differences in the power to predict IBD type.^39,40^ Olaisen et al. found that the microbiota associated with inflamed and proximal uninflamed ileal biopsies collected during the same endoscopic examination were undifferentiable based on compositional patterns and that IBD type could not be differentiated based on ileal mucosal microbiota composition.^26^ This raises the question of why the composition of the mucosal-associated microbiota cannot differentiate between disease presentations that can be clearly identified using the stool microbiota. In a study by Vaga et al.,^36^ individual intestinal mucosal biopsies were associated with a microbiota decrease in bacterial richness relative to fecal microbiota. When microbiota profiles from multiple colonic mucosal samples were pooled for each individual, they observed a significantly higher bacterial richness, but differences between pooled intestinal mucosal and stool microbiota persisted.^36^ This may be explained by the high bacterial concentration in the stool, making subtle differences easier to detect.

Alpha diversity measures were not significantly different between groups for multiple factors evaluated but differed significantly based on IBD type, whether a sample was obtained from inflamed tissue and sex. Similarly to our findings, it was previously reported that samples taken from healthy tissue in those with CD have lower alpha diversity than those taken from UC counterparts.^41^ In the LOCATION cohort, alpha diversity was significantly lower in those with CD and IC relative to UC. Given that the overall composition does not vary significantly between locations, variation in a small subset of species is likely responsible for differences in beta diversity. IC patients are rarely included in studies, and we found they display similar patterns in alpha and beta diversity to individuals with CD as opposed to UC. Further, IC patients had an increased prevalence of IAA, similar to CD patients, indicating that the overall IC phenotype aligns more closely with CD. Overall, these results suggest that higher variation in a small number of species in the microbiota might be more informative than global structural patterns, and small differences in beta diversity across the colon may be a signature of intestinal dysbiosis.

Multiple taxa were associated with the IAA. *D. longicatena, L. ammoniilytica*, and *R. torques* groups were found to be lower in those with IAA. *D. longicatena*, in particular, was also lower in those with CD specifically, which is interesting given the higher rate of IAA in CD. Relative to healthy controls, lower *Dorea* spp. relative abundance has been observed in fecal samples from CD patients^42^ and mucosal samples from UC patients,^41^ with higher relative abundance in the mucosa following treatment and disease remission.^43^ However, higher *Dorea* relative abundance at the genus level is associated with relatively higher levels of mucosal pro-inflammatory cytokines,^44^ indicating *D. longicatena* may harbor unique features compared to other *Dorea* that confer a protective benefit. Similarly, lower abundances of *D. longicatena* are observed in ankylosing spondylitis (AS),^45^ a form of SpA that occurs more frequently in IBD, leading us to speculate that suppression of mucosal inflammation by *D. longicatena* may have systemic effects. This association might be potentiated by the known production of indole-3-acetate by *D. longicatena*, a tryptophan metabolite that reduces inflammation via aryl hydrocarbon receptor activation.^46^

In a sex-specific manner, *R. intestinalis* and *Corynebacterium* were uniquely differentially abundant in those with versus without IAA. In several studies, *R. intestinalis*, which was higher in females without IAA, was observed to have a lower relative abundance in IBD, particularly during active disease.^23,47^ *R. intestinalis* is also observed in lower abundance in AS, particularly in the context of active disease and inflammation,^48^ and similar metabolic functional pathways were found to be enriched as previously observed in IBD.^49^ *R. intestinalis* has been associated with amelioration of colitis, lower disease activity indices, and reduced expression of oncostatin M (which downregulates tight junction protein expression) and the pro-inflammatory cytokine TNF-α in 2,4,6-trinitrobenzene-sulfonic-acid (TNBS) – and dextran sodium sulfate (DSS) – induced colitis models.^50,51^ Along with *Faecalibacterium prausnitzii*, *Clostridium leptum*, and *Eubacterium rectale*,^52^ *Roseburia* produces large amounts of short-chain fatty acids (SCFAs) in the gut, particularly butyrate.^53^ Butyrate harbors anti-inflammatory effects, ameliorating colitis and modulating tight junction integrity,^54,55^ and lower levels of butyrate producers are associated with decreased response to sulfasalazine for IAA.^56^ Given the altered barrier integrity observed in IBD and SpA,^57,58^ protection of barrier integrity via modulation of butyrate levels is a hypothesized mechanism by which *R. intestinalis* could be protective.

In a TNBS-induced colitis murine model, administration of *R. intestinalis* via intragastric gavage ameliorated colonic disease-associated increases in IL-17 expression and histopathologic inflammation.^59^ Butyrate, which *R. intestinalis* produces, can dampen IL-17 production, which has been demonstrated to ameliorate colitis in rat models.^55^ IL-17 expression plays a role in mucosal pathogen response, and while IL-17 is needed for appropriate tight junction formation,^60,61^ it is over-expressed in IBD, psoriasis, and SpA;^62,63^ aberrant IL-17 expression (either over- or under-expression) is associated with altered permeability,^64,65^ and while IL-17 blockade via therapies like secukinumab are commonly used for non-IBD SpA, it can paradoxically initiate or worsen preexisting IBD.^66^ In several systemic, non-GI diseases, IL-17 has been shown to be elevated in the context of LPS-induced inflammation,^67,68^ suggesting butyrate production by *R. intestinalis* may be involved in modulating mucosal IL-17 levels. *R. intestinalis* also plays direct roles in modulating other mechanisms of immune response; for instance, in a TNBS murine model pretreated with *R. intestinalis*, macrophage mucosal infiltration is decreased, as are IL-6 and STAT3 production, in association with a decrease in SCFAs.^69^ *R. intestinalis* also mediates TLR5 stimulation of intestinal epithelial cells, increasing IL-10 production.^70^ Thus, *R. intestinalis* plays roles in upregulating anti-inflammatory and downregulating pro-inflammatory pathways. *R. intestinalis* butyrate production and associated improvements in mucosal barrier integrity may contribute to decreased systemic symptoms in those with a higher abundance of *R. intestinalis*. However, additional multiple metabolites and immune mechanisms are also likely involved.

However, the question of sex-specific effects remains. In our cohort, *R. intestinalis* levels were the same in males and females when participants were not sub-stratified by joint EIM status. Although AS and axial involvement in SpA are exceptions,^71^ females are generally more likely to have IBD-associated^7^ and other forms of inflammatory arthritis.^72,73^ Interestingly, IL-17-related inflammation is thought to be differentially regulated in males and females, not only in inflammatory arthritis^71^ but also in other related autoimmune diseases such as multiple sclerosis.^74^ Estrogen deficiency has been shown to stimulate Th17 differentiation and IL-17 as well as STAT3 production.^75^ In contrast, dydrogesterone, a synthetic progestin, has been shown to suppress IL-17 production,^76^ suggesting potential steroid hormone-mediated differential regulation. If *R. intestinalis* is differentially regulating or contributing to altered IL-17 expression in females, this could explain the sex-specific differences observed.

*Corynebacterium* also showed sex-specific differences, though in an opposing pattern, with males with IAA showing a higher relative abundance than their male counterparts without joint EIMs. Higher relative abundances of *Corynebacterium* were previously associated with IBD and SpA,^12^ as well as more complicated IBD.^77,78^ *Corynebacterium* is significantly enriched in scrapings from anorectal fistulas,^77^ and in stool samples from those with PSC and IBD compared to IBD alone.^78^ Numerous cases of *Corynebacterium-*associated with septic arthritis are reported.^79,80^ Furthermore, in a rat model, the intrathermal introduction of the purified cell wall of *Corynebacterium rubrum* has been shown to induce severe, progressive arthritis.^81^ Thus, *Corynebacterium* spp. appear to be highly pro-inflammatory, and higher relative abundance could contribute to arthritis by inducing mucosal inflammation, leading to a leakier mucosal barrier and/or production of metabolites mediating joint inflammation. With regard to sex, *Corynebacterium* has been shown to increase testosterone and IL-6 production in animal models^82^and can metabolize testosterone into multiple byproducts.^83^ Thus, steroid hormone differences may drive some underlying sex-specific differences observed in participants with EIMs.

This study has many strengths. First, using intestinal biopsy samples affords an assessment of the mucosa-associated microbiota, which is most likely to affect the intestinal barrier. Further, the deep phenotyping of the cohort, particularly the classifications of joint manifestations, allows for group sub-stratification and in-depth analyses. We administered comprehensive questionnaires to participants regarding joint manifestations and worked with a board-certified rheumatologist to classify joint findings, assuring participants were accurately classified. The cohort size, particularly the sub-population with IAA, is relatively large and more racially diverse than most equivalently sized IBD cohorts; given the high number of patients on biologics in both groups, its potential to confound results is limited.

Nonetheless, our work has limitations. Our study was not adequately powered to deeply assess differences in participants with IAA with active joint involvement, nor to compare peripheral vs axial involvement. Sub-stratification by sex and IAA resulted in smaller sample subgroup sizes (*n* = 19 and 62 for men with or without IAA, and 32 and 41 for women with or without IAA, respectively). For most participants, we could not obtain complete data on locations of historic colon involvement (*i.e*., right colon, transverse, left colon, rectum), limiting our ability to assess the involvement of specific disease locations within the colon on EIMs. We also acknowledge that colonoscopy bowel cleansing may alter mucosal microbiota composition; however, it would be challenging to distinguish how bowel preparation may impact intestinal mucosa-associated microbiome composition. 16S rRNA gene amplicon sequencing was used to characterize the composition of the intestinal mucosal microbiota, which has lower taxonomic resolution than shotgun metagenomic sequencing (albeit affording deeper taxonomic sampling), and cannot be used to assess strain-level differences or functional capabilities of the microbiome (based on gene presence). Additionally, data on factors that may influence sex-specific results, such as menopausal status and hormone levels or use of hormonal medications, were not collected and could not be evaluated.

Altogether, we found differences in the intestinal mucosa-associated microbiota between those with and without IAA, many of which were sex-specific. In particular, we identified changes at the mucosal level, which are paramount for developing targeted microbiome-based therapeutics, as mucosal and fecal sample beta diversity is high for individuals with IBD,^40,84,85^ and potential targets may be over- or under-represented in stool studies.^38,86^ Whereas our findings are correlative, longitudinal follow-up of this cohort will provide additional strength to these observations. At the same time, multi-omic approaches (immunological, transcriptomics, and metabolomics) could offer a mechanistic understanding of the impact of the mucosa-associated microbiome on the gut epithelium and in the development and progression of EIMs. Specifically, tandem samples were collected for metabolomic analysis at the same time as samples used for microbiota analysis to characterize potential metabolite targets associated with identified microbiota. *In vitro* validation studies using 3D-organotypic models could validate these proposed mechanisms, including how the microbiota disrupts intestinal permeability (IP). Introducing previously identified metabolites or probiotic therapies (*e.g.*, containing *R. intestinalis*) to an organ-on-chip model system and studying effects on IP is a necessary first step before testing these treatments on animal and, eventually, human models. Incorporating hormone treatment into experiments can help shed light on the causal effects of sex-specific hormonal differences on modulation of the microbiome and subsequent impact to IP. Understanding the effect of IP is key to developing targeted biotherapeutic products tailored to supplement missing microbes, producing specific metabolites that could help restore IP and intestinal homeostasis in patients suffering from IAA.

## Supplementary Material

Supplemental Material

## Data Availability

Deidentified metadata for the study is provided in the supplemental materials. Taxonomic data used for analysis are provided as a supplemental table and supporting sequence data is available under SRA accession number PRJNA1128669. R code used to analyze data and generate figures can be accessed at the laboratory GitHub (https://github.com/ravel-lab/LOCATION-IBD).
