# Supplementary material for: The intestinal mucosa-associated microbiota in IBD-associated arthritis displays lower relative abundance of Roseburia intestinalis
Source: Gut Microbes. 2025 May 18;17(1):2505114. doi: 10.1080/19490976.2025.2505114 (PMC12087651; doi:10.1080/19490976.2025.2505114)
Supplement: Supplemental Material [file KGMI_A_2505114_SM3174.zip › supplementarymaterialsAlizadeh.docx]

**Supplementary materials:
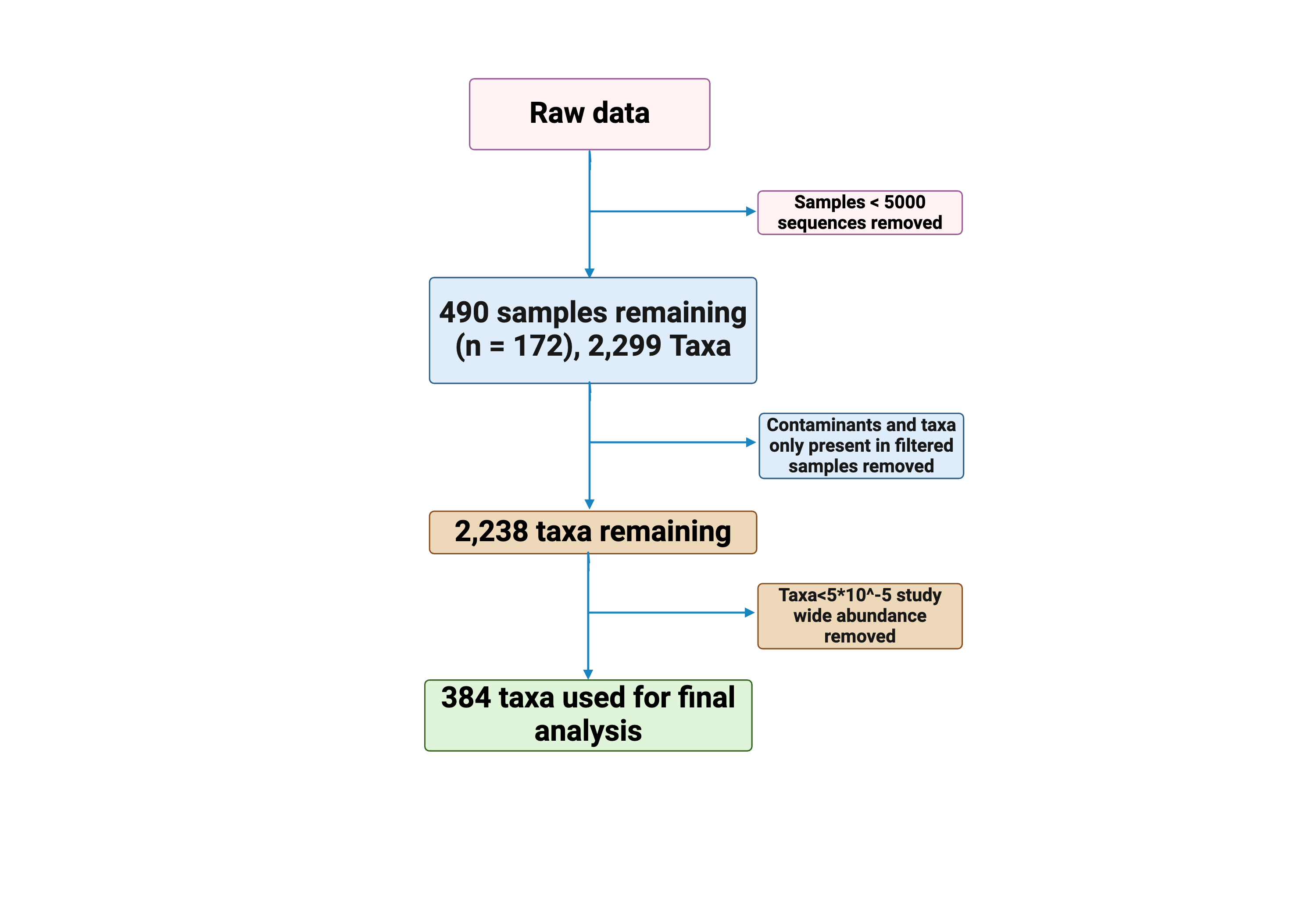
**

Supplemental Figure 1. Pipeline of data processing used to arrive at the final dataset.

**
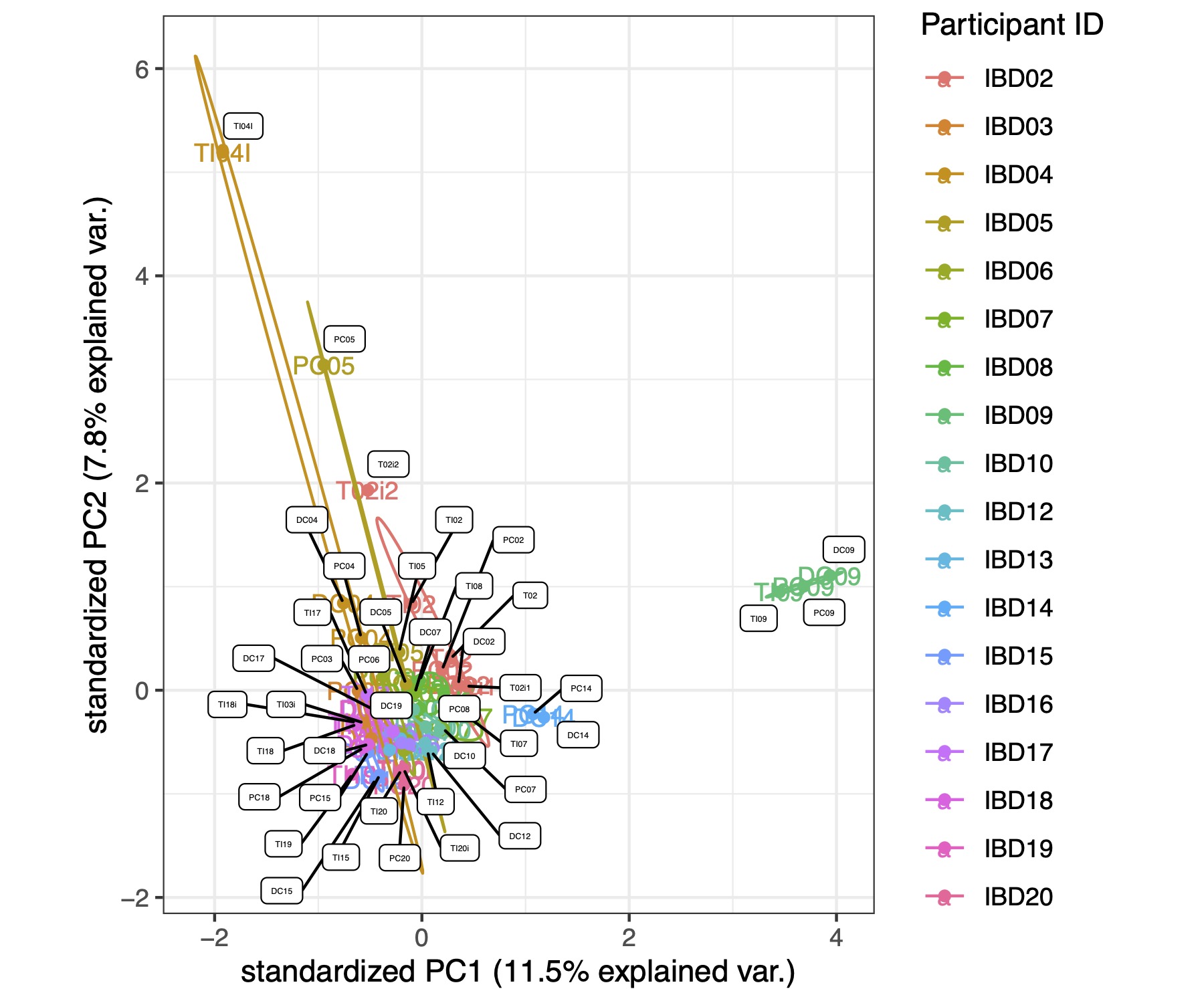
**

Supplemental Figure 2. PCA of a subset of intestinal mucosal microbiota labeled by sample IDs, for the first 20 participants. Points are color coded by participant ID.

**
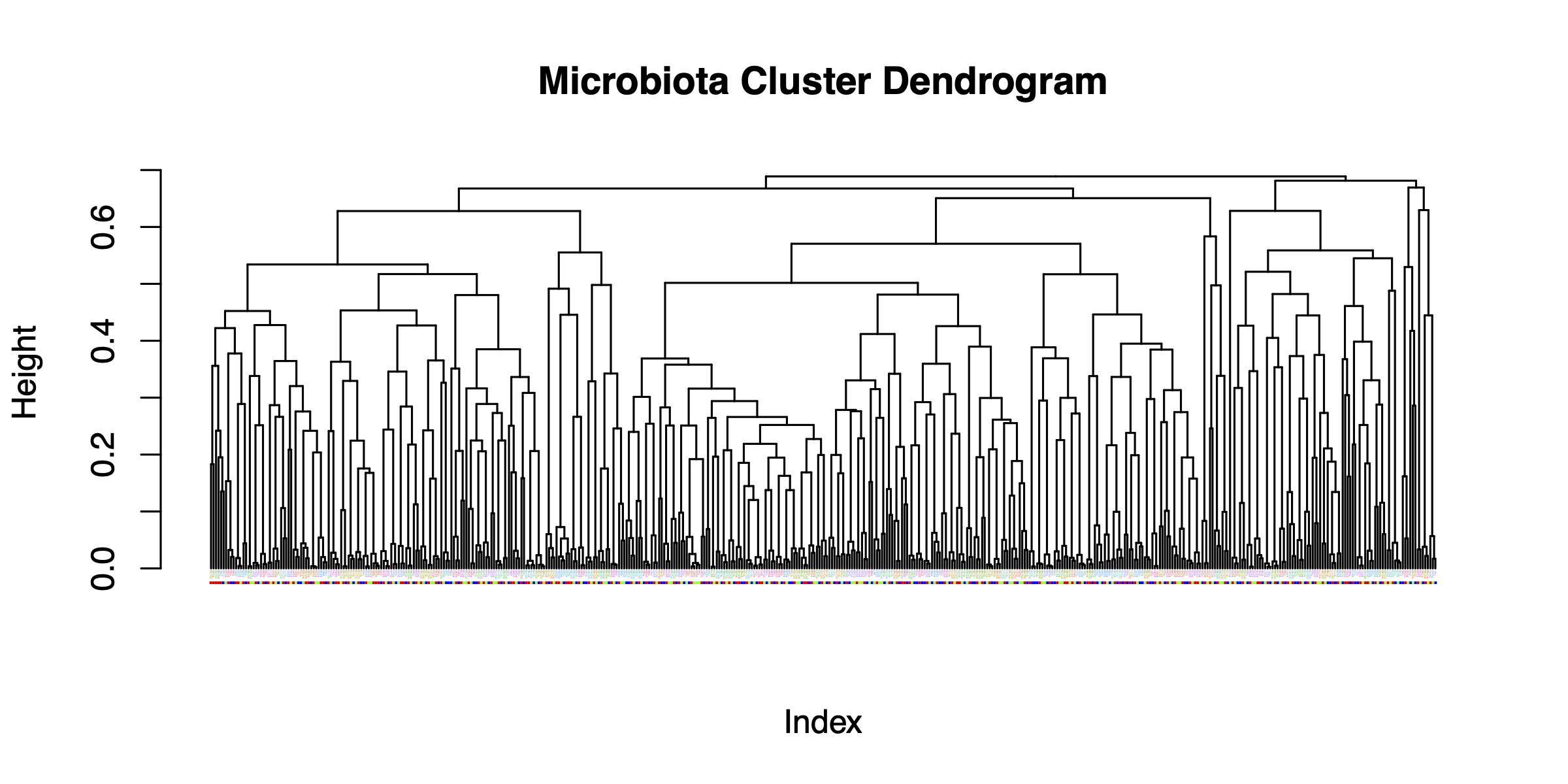
**

Supplemental Figure 3. Hierarchical clustering of the composition of the intestinal mucosal microbiota from all samples analyzed. Euclidean distances and Ward linkage were used for clustering. Color coding on the bottom row indicates sampling location, where red = distal descending colon, green = hepatic flexure, and blue = terminal ileum.

Supplemental Figure 4. Distribution of Jensen Shannon Distances (JSD) between each intestinal mucosal microbiota analyzed.

Supplemental Figure 5. Distribution of Shannon indices across all samples analyzed.

Supplemental Figure 7. Distribution of mean Jensen Shannon Distances (JSD) between intestinal mucosal microbiota from each participant in the study. JSDs between each site were calculated and averaged for each participant.

Supplemental Table 2. A comparison of bacterial taxa relative abundance in those with or without IBD-associated arthritis. Comparisons were performed using ALDEx2 and a t-test. A positive difference indicates a higher bacterial taxa relative abundance in those with joint EIMs, while a negative difference indicates higher bacterial taxa relative abundance in those without joint EIMs.

|  | **Difference** | **Dispersion** | **Effect** | **Welch’s t-test q-value** | **Wilcoxon Rank test q-value** |
| --- | --- | --- | --- | --- | --- |
| ***Ruminococcus torques* group** | -0.801 | 3.807 | -0.162 | 0.1729 | 0.0751 |
| ***D. longicatena*** | -1.706 | 9.206 | -0.193 | 0.0499 | 0.0509 |
| ***Anaerococcus*** | 1.222 | 6.028 | 0.188 | 0.1748 | 0.0957 |
| ***Corynebacterium*** | 1.326 | 5.197 | 0.225 | 0.0308 | 0.0170 |
| ***R. intestinalis*** | -1.699 | 7.376 | -0.224 | 0.0321 | 0.0562 |
| ***S. intestinalis*** | 1.580 | 6.801 | 0.222 | 0.0547 | 0.0452 |
| ***C. accolens*** | 1.413 | 7.980 | 0.179 | 0.0885 | 0.1109 |
| ***Lawsonella*** | 1.500 | 6.813 | 0.212 | 0.0906 | 0.0488 |
| ***Moraxella*** | 1.629 | 6.802 | 0.237 | 0.0637 | 0.0367 |

Supplemental Table 3. Assessment of the relationship between bacterial taxa relative abundance and joint EIMs, while controlling for multiple potential confounders. Both multiple GLMs and mixed multiple GLMs were performed. The generalized base equation for each was the following: joint_EIM_status ~ CLR_transformed_relative_abundance_taxa + IBD_type + sampling_location + inflamed_site. An OR > 1 indicates a relative increase in those with joint EIMs.

|  | **OR** | **CI (95%)** | **p-value** | **q-value** |
| --- | --- | --- | --- | --- |
| **Multiple GLM** |  |  |  |  |
| ***Butyricicoccus*** | 1.525 | [1.232,1.900] | 0.000126 | 0.014777 |
| ***R. intestinalis*** | 0.411 | [0.266,0.600] | 0.0000164 | 0.005750 |
| ***R. hominis*** | 1.991 | [1.361, 2.978] | 0.000539 | 0.047237 |
| ***Frisingicoccus*** | 2.403 | [1.463, 4.155] | 0.000930 | 0.065205 |
| ***L. ammoniilytica*** | 0.534 | [0.387, 0.728] | 0.0000894 | 0.014777 |
| **Mixed Multiple GLM** |  |  |  |  |
| ***Butyricicoccus*** | 1.525 | NA | 0.000294 | 0.034304 |
| ***R. intestinalis*** | 0.411 | NA | 0.0000522 | 0.018301 |
| ***R. hominis*** | 1.991 | NA | 0.000539 | 0.047221 |
| ***Frisingicoccus*** | 2.403 | NA | 0.000930 | 0.065183 |
| ***L. ammoniilytica*** | 0.534 | NA | 0.0000211 | 0.034304 |

Supplemental Table 4. Statistical analysis of the relationship between bacterial taxa relative abundance and joint EIMs, controlling for sex, IBD type, and the interaction between each of these factors and bacterial taxa relative abundance. Modeling was performed using multiple GLMs (OR from mixed GLMs in parentheses). An OR > 1 indicates a relative increase in individuals with joint EIMs. Each set of grouped factors represents one model

|  | OR | | CI (95%) | p-value |
| --- | --- | --- | --- | --- |
| IC (relative to CD) | | 1.969 (1.969) | [0.753,5.046] | 0.156712 (0.156711) |
| UC (relative to CD) | | 0.257 (0.257) | [0.120,0.498] | 0.000155 (0.000155) |
| Male (relative to Female sex) | | 0.345 (0.345) | [0.208,0.562] | 0.000250 (0.000250) |
| *Butyricicoccus* | | 1.263 (1.263) | [0.931,1.730] | 0.138460 (0.138460) |
| Interaction between male sex and *Butyricicoccus* (relative to female sex) | | 1.487 (1.487) | [0.968,2.305] | 0.071926 (0.024845) |
| Interaction between IC and *Butyricicoccus* (relative to CD) | | 1.296 (1.296) | [0.498,3.925] | 0.612912 (0.612912) |
| Interaction between UC and *Butyricicoccus* (relative to CD) | | 1.091 (1.091) | [0.676,1.795] | 0.725724 (0.725724) |
|  | |  |  |  |
| IC (relative to CD) | | 1.779 (1.791) | [0.737,4.345] | 0.197807 (0.202499) |
| UC (relative to CD) | | 0.293 (0.291) | [0.159,0.513] | 0.000035 (0.000828) |
| Male (relative to Female sex) | | 0.383 (0.379) | [0.245,0.593] | 0.0000207 (0.000854) |
| *R. intestinalis* | | 0.182 (0.180) | [0.064,0.393] | 0.000182 (0.000373) |
| Interaction between male sex and *R. intestinalis* (relative to female sex) | | 3.124 (3.151) | [1.254,9.297] | 0.022680 (0.024845) |
| Interaction between IC and *R. intestinalis* (relative to CD) | | 1.934 (1.944) | [0.055,24.09] | 0.656510 (0.657472) |
| Interaction between UC and *R. intestinalis* (relative to CD) | | 1.084 (1.085) | [0.379,3.270] | 0.881756 (0.881153) |
|  | |  |  |  |
| IC (relative to CD) | | 2.141 (2.141) | [0.890,5.255] | 0.088755 (0.088755) |
| UC (relative to CD) | | 0.289 (0.289) | [0.151,0.518] | 0.000070 (0.000071) |
| Male (relative to Female sex) | | 0.445 (0.445) | [0.284,0.690] | 0.000333 (0.000333) |
| *R. hominis* | | 2.019 (2.019) | [1.080,4.088] | 0.036154 (0.036153) |
| Interaction between male sex and R. hominis (relative to female sex) | | 0.924 (0.924) | [0.392,2.154] | 0.855519 (0.855523) |
| Interaction between IC and *R. hominis (*relative to CD) | | 0.181 (0.181) | [0.009,1.631] | 0.162079 (0.162081) |
| Interaction between UC and *R. hominis* (relative to CD) | | 1.211 (1.211) | [0.470,3.249] | 0.695466 (0.695497) |
|  | |  |  |  |
| IC (relative to CD) | | 2.615 (2.626) | [1.044,6.866] | 0.042177 (0.046633) |
| UC (relative to CD) | | 0.318 (0.317) | [0.174,0.553] | 0.000094 (0.000196) |
| Male (relative to Female sex) | | 0.459 (0.458) | [0.294,0.711] | 0.000536 (0.000997) |
| *Frisingicoccus* | | 1.801 (1.804) | [0.951,3.780] | 0.087796 (0.091539) |
| Interaction between male sex and *Frisingicoccus* (relative to female sex) | | 3.242 (3.258) | [0.758,20.09] | 0.153695 (0.157713) |
| Interaction between IC and *Frisingicoccus* (relative to CD) | | 269.5 (274.8) | [0.108,NA] | 0.521579 (0.523460) |
| Interaction between UC and *Frisingicoccus* (relative to CD) | | 0.446 (0.444) | [0.069,2.009] | 0.339027 (0.341846) |
|  | |  |  |  |
| IC (relative to CD) | | 2.207 (2.207) | [0.895,5.501] | 0.083444 (0.083444) |
| UC (relative to CD) | | 0.256 (0.256) | [0.121,0.494] | 0.000127 (0.000127) |
| Male (relative to Female sex) | | 0.483 (0.483) | [0.301,0.769] | 0.002354 (0.002354) |
| *L. ammoniilytica* | | 0.383 (0.383) | [0.208,0.653] | 0.000921 (0.000921) |
| Interaction between male sex and *L. ammoniilytica* (relative to female sex) | | 1.627 (1.627) | [0.858,3.217] | 0.145413 (0.145415) |
| Interaction between IC and *L. ammoniilytica* (relative to CD) | | 1.576 (1.576) | [0.447,5.204] | 0.438784 (0.438785) |
| Interaction between UC and *L. ammoniilytica* (relative to CD) | | 1.022 (1.022) | [0.483,2.224] | 0.955545 (0.955546) |
|  | |  |  |  |
| IC (relative to CD) | | 2.054 (2.054) | [0.373,12.87] | 0.41023 (0.41023) |
| UC (relative to CD) | | 0.130 (0.130) | [0.027,0.487] | 0.00533 (0.00533) |
| Male (relative to Female sex) | | 0.519 (0.519) | [0.225,1.714] | 0.11814 (0.11814) |
| *Ruminococcus Torques* group | | 0.883 (0.883) | [0.718,1.080] | 0.22778 (0.22778) |
| Interaction between male sex and *Ruminococcus Torques* group (relative to female sex) | | 0.944 (0.944) | [0.710,1.257] | 0.69314 (0.69314) |
| Interaction between IC and *Ruminococcus Torques* group (relative to CD) | | 0.991 (0.991) | [0.506,1.895] | 0.97815 (0.97815) |
| Interaction between UC and *Ruminococcus Torques* group (relative to CD) | | 1.416 (1.416) | [0.924,2.260] | 0.12424 (0.12424) |
|  | |  |  |  |
| IC (relative to CD) | | 1.625 (1.627) | [0.561,4.801] | 0.36368 (0.36552) |
| UC (relative to CD) | | 0.278 (0.277) | [0.121,0.575] | 0.00113 (0.00168) |
| Male (relative to Female sex) | | 0.434 (0.433) | [0.261,0.714] | 0.00188 (0.00111) |
| *D*. *longicatena* | | 0.820 (0.819) | [0.597,1.115] | 0.20986 (0.21619) |
| Interaction between male sex and *D. longicatena* (relative to female sex) | | 1.018 (1.018) | [0.698,1.481] | 0.92766 (0.92672) |
| Interaction between IC and *D. longicatena* (relative to CD) | | 1.412 (1.414) | [0.621,3.280] | 0.40430 (0.40637) |
| Interaction between UC and *D. longicatena* (relative to CD) | | 1.270 (1.271) | [0.800,2.021] | 0.30993 (0.31294) |
|  | |  |  |  |
| IC (relative to CD) | | 1.504 (1.504) | [0.529,4.263] | 0.436291 (0.437318) |
| UC (relative to CD) | | 0.317 (0.317) | [0.158,0.602] | 0.000718 (0.001096) |
| Male (relative to Female sex) | | 0.350 (0.350) | [0.202,0.597] | 0.000139 (0.000308) |
| *Anaerococcus* | | 1.129 (1.129) | [0.842,1.523] | 0.420377 (0.422219) |
| Interaction between male sex and *Anaerococcus* (relative to female sex) | | 0.796 (0.796) | [0.540,1.175] | 0.248150 (0.251010) |
| Interaction between IC and *Anaerococcus* (relative to CD) | | 0.636 (0.636) | [0.236,1.495] | 0.319503 (0.322334) |
| Interaction between UC and *Anaerococcus* (relative to CD) | | 0.961 (0.961) | [0.604,1.568] | 0.868214 (0.868229) |
|  | |  |  |  |
| IC (relative to CD) | | 2.211 (2.211) | [0.868,6.283] | 0.10777 (0.10777) |
| UC (relative to CD) | | 0.218 (0.218) | [0.106,0.411] | 0.000009 (0.000009) |
| Male (relative to Female sex) | | 0.521 (0.521) | [0.328,0.824] | 0.00546 (0.00546) |
| *Corynebacterium* | | 1.121 (1.121) | [0.907,1.393] | 0.29269 (0.29269) |
| Interaction between male sex and *Corynebacterium* (relative to female sex) | | 1.425 (1.425) | [1.035,1.988] | 0.03286 (0.03286) |
| Interaction between IC and *Corynebacterium* (relative to CD) | | 1.079 (1.079) | [0.606,2.125] | 0.80637 (0.80637) |
| Interaction between UC and *Corynebacterium* (relative to CD) | | 0.506 (0.506) | [0.321,0.782] | 0.00259 (0.00259) |
|  | |  |  |  |
| IC (relative to CD) | | 1.513 (1.513) | [0.868,3.857] | 0.384902 (0.384902) |
| UC (relative to CD) | | 0.354 (0.354) | [0.196,0.613] | 0.000336 (0.000336) |
| Male (relative to Female sex) | | 0.450 (0.450) | [0.286,0.703] | 0.000488 (0.000488) |
| *C. accolens* | | 1.904 (1.904) | [1.002,3.825] | 0.056407 (0.056407) |
| Interaction between male sex and *C. accolens* (relative to female sex) | | 0.856 (0.856) | [0.298,2.445] | 0.769249 (0.769250) |
| Interaction between IC and *C. accolens* (relative to CD) | | 2.4*10^9^  (3.9*10^23^) | [1.59*10^-36^,NA] | 0.981212 (0.999998) |
| Interaction between UC and *C. accolens* (relative to CD) | | 0.745 (0.745) | [0.321,2.978] | 0.694410 (0.694411) |
|  | |  |  |  |
| IC (relative to CD) | | 2.550 (2.550) | [0.976,7.184] | 0.061581 (0.061583) |
| UC (relative to CD) | | 0.283 (0.282) | [0.144,0.522] | 0.000113 (0.000113) |
| Male (relative to Female sex) | | 0.426 (0.426) | [0.261,0.688] | 0.000547 (0.000547) |
| *Lawsonella* | | 1.083 (1.083) | [0.803,1.470] | 0.604943 (0.604944) |
| Interaction between male sex and *Lawsonella* (relative to female sex) | | 0.976 (0.976) | [0.631,1.525] | 0.912172 (0.912172) |
| Interaction between IC and *Lawsonella* (relative to CD) | | 2.564 (2.564) | [1.019,11.32] | 0.092948 (0.092963) |
| Interaction between UC and *Lawsonella* (relative to CD) | | 0.766 (0.766) | [0.458,1.319] | 0.317895 (0.317896) |
|  | |  |  |  |
| IC (relative to CD) | | 2.378 (2.382) | [0.952,6.063] | 0.063510 (0.067826) |
| UC (relative to CD) | | 0.326 (0.326) | [0.172,0.586] | 0.000310 (0.000507) |
| Male (relative to Female sex) | | 0.336 (0.335) | [0.208,0.535] | 0.0000059 (0.000026) |
| *Moraxella* | | 1.010 (1.010) | [0.632,1.627] | 0.966360 (0.966391) |
| Interaction between male sex and *Moraxella* (relative to female sex) | | 0.458 (0.457) | [0.242,0.847] | 0.013880 (0.015965) |
| Interaction between IC and *Moraxella* (relative to CD) | | 2.097 (2.099) | [0.382,13.31] | 0.367620 (0.369257) |
| Interaction between UC and *Moraxella* (relative to CD) | | 1.060 (1.060) | [0.536,2.148] | 0.867920 (0.867742) |

Supplemental Table 5. Evaluation of the relationship between joint EIMs and *R. intestinalis* quartile, controlling for sex, IBD type, and the interaction between *R. intestinalis* quartile and sex. Modeling was performed using mixed GLMs, and quartile was a binary variable, indicating whether a sample had a CLR transformed relative abundance of *R. intestinalis* greater than or below the threshold for 4^th^ quartile in all samples. An OR > 1 indicates a relative higher relative abundance of *R. intestinalis* in individuals with joint EIMs.

|  | **OR** | **p-value** |
| --- | --- | --- |
| **Being in the 4^th^ quartile of *R. intestinalis* abundance** | 0.688 | 0.335625 |
| **Male sex (relative to female)** | 0.330 | 0.000045 |
| **IC (relative to CD)** | 1.696 | 0.243315 |
| **UC (relative to CD)** | 0.346 | 0.000461 |
| **The interaction between *R. intestinalis* quartile and female sex (relative to not being above the threshold/male sex)** | 0.207 | 0.011711 |

Supplemental Table 6. Assessment of the relationship between taxa abundance and IBD type, while controlling for multiple potential confounders. An OR > 1 indicates a relative increase in those with CD compared to those with UC.

| Taxa | OR | CI (95%) | p-value | q-value |
| --- | --- | --- | --- | --- |
| *Faecalibacterium prausnitzii* | 1.822 | [1.506,2.261] | 0.000000 | 0.000002 |
| *Escherichia Shigella* | 0.709 | [0.592,0.832] | 0.000068 | 0.002554 |
| *Ruminococcus torques* group | 1.403 | [1.112,1.793] | 0.005340 | 0.046814 |
| *Ruminococcus gnavus* group | 0.705 | [0.576,0.848] | 0.000376 | 0.008339 |
| *Faecalibacterium* | 1.623 | [1.340,1.994] | 0.000002 | 0.000205 |
| *Dorea* | 0.621 | [0.473,0.788] | 0.000225 | 0.006054 |
| *Bacteroides massiliensis* | 1.503 | [1.202,1.897] | 0.000409 | 0.008558 |
| *Subdoligranulum* | 2.106 | [1.641,2.770] | 0.000000 | 0.000004 |
| *Fusobacterium* | 0.645 | [0.472,0.853] | 0.003626 | 0.040202 |
| *Alistipes putredinis* | 1.678 | [1.305,2.187] | 0.000079 | 0.002719 |
| *Veillonella* | 0.631 | [0.481,0.800] | 0.000369 | 0.008339 |
| *Eubacterium hallii* group | 1.550 | [1.187,2.051] | 0.001630 | 0.024578 |
| *Collinsella aerofaciens* | 1.722 | [1.305,2.306] | 0.000169 | 0.004891 |
| *Coprococcus comes* | 1.824 | [1.392,2.421] | 0.000019 | 0.000873 |
| *Dorea longicatena* | 2.143 | [1.551,3.023] | 0.000007 | 0.000663 |
| *Phascolarctobacterium faecium* | 1.670 | [1.273,2.232] | 0.000321 | 0.008063 |
| *Fusicatenibacter saccharivorans* | 1.484 | [1.135,1.954] | 0.004280 | 0.042462 |
| *Megasphaera* | 0.666 | [0.509,0.851] | 0.001887 | 0.026353 |
| *Dorea formicigenerans* | 2.005 | [1.444,2.824] | 0.000044 | 0.001848 |
| *Blautia faecis* | 1.611 | [1.192,2.210] | 0.002359 | 0.029640 |
| *Erysipelatoclostridium ramosum* | 0.624 | [0.471,0.807] | 0.000571 | 0.011324 |
| *Fusobacterium nucleatum* | 0.619 | [0.430,0.846] | 0.005295 | 0.046814 |
| UCG.002 | 2.157 | [1.541,3.093] | 0.000014 | 0.000863 |
| *Anaerostipes* | 0.536 | [0.352,0.776] | 0.001861 | 0.026353 |
| *Colidextribacter* | 1.597 | [1.219,2.124] | 0.000900 | 0.016160 |
| *Lachnospiraceae UCG010* | 1.612 | [1.213,2.180] | 0.001342 | 0.023005 |
| *Coprococcus catus* | 2.605 | [1.720,4.117] | 0.000016 | 0.000863 |
| *Alistipes finegoldii* | 2.135 | [1.418,3.433] | 0.000691 | 0.013026 |
| *Lachnospiraceae UCG009* | 0.543 | [0.346,0.830] | 0.000009 | 0.000683 |
| *Bacteroides ovatus* | 2.449 | [1.681,3.724] | 0.001612 | 0.024578 |
| *Oscillibacter* | 1.973 | [1.314,3.067] | 0.002339 | 0.029640 |
| UCG.003 | 1.719 | [1.223,2.464] | 0.001976 | 0.026610 |
| *Dialister* | 1.705 | [1.233,2.439] | 0.004891 | 0.044971 |
| *Ruminococcus bicirculans* | 0.510 | [0.313,0.804] | 0.001623 | 0.024578 |
| *GCA.900066575* | 0.465 | [0.287,0.750] | 0.003138 | 0.038165 |
| *Sphingomonas* | 0.608 | [0.435,0.844] | 0.004734 | 0.044620 |
| *Eisenbergiella tayi* | 0.491 | [0.297,0.802] | 0.000137 | 0.004319 |
| *Merdibacter* | 0.352 | [0.195,0.581] | 0.003613 | 0.040202 |
| *Hungatella* | 0.399 | [0.200,0.709] | 0.003934 | 0.041199 |
| *Gemella morbillorum* | 0.582 | [0.399,0.839] | 0.003897 | 0.041199 |
| *Adlercreutzia equolifaciens* | 0.376 | [0.180,0.690] | 0.003298 | 0.038858 |
| *Eubacterium fissicatena* group | 0.579 | [0.397,0.828] | 0.004545 | 0.043931 |
| *Candidatus Soleaferrea* | 0.654 | [0.486,0.877] | 0.004045 | 0.041211 |
| *Anaerotruncus colihominis* | 0.518 | [0.327,0.810] | 0.000000 | 0.000002 |
